# The National Medical Union

**Published:** 1914-01-17

**Authors:** 


### THE PROFESSION OF MEDICINE.

### The National Medical Union.

### The Sussex Non-Panel Union.

The Non-Panel Organisation for Brighton and East Sussex, at a meeting on the 6th instant, was dissolved. On Thursday, January 8, a meeting of non-panel practitioners was held at Dr. Greenyer's house, when the Sussex Non-Panel Union was started. It will federate with the National Medical Union. The annual subscription is 25s., of which 21s. is to be sent to the Treasurer of the National Medical Union for each member.

The area covered will be the whole of Sussex. It is proposed to have meetings at convenient centres in the county.

### The Huddersfield Non-Panel Association.

A meeting of the Huddersfield Non-Panel Association was held on December 30, 1913. Dr. Marshall (Chairman of the Association) presided.

A discussion took place upon the future of the Association, and eventually, on the motion of Dr. Williams, seconded by Dr. Crosland, it was decided unanimously that the Huddersfield Non-Panel Association federate with the National Medical Union, and that the Secretary communicate with other Non-Panel Associations and individual non-panel doctors in Yorkshire with a view to the formation of a Yorkshire Branch of the Union.

The officers of the Association were re-elected—namely, Chairman: Dr. Marshall; Secretary and Treasurer: Dr. R. H. Trotter, Holmfirth, Huddersfield; Committee: Drs. Williams, Crosland, and McCully, in addition to the Chairman and Secretary. The annual subscription to the local division was fixed at one shilling a member, in addition to one guinea for the National Medical Union. Dr. Trotter will be glad to hear from secretaries of other Yorkshire Non-Panel Associations, or from individual non-panel doctors in Yorkshire, so that arrangements may be made for forming a Yorkshire Branch of the National Medical Union.

### A Question of Organisation.

In a letter from Dr. Trotter, it is suggested that "to make the divisions of the new National Medical Union conterminous with the Insurance areas would be a mistake in some parts of the country at all events. For instance, we in Holmfirth are six miles from Huddersfield, which is a natural centre for us medically; but Huddersfield is a county borough, with an Insurance Committee of its own, whereas we are in the West Riding Insurance area, which also includes, for example, places near Sheffield, twenty miles south of us, and places near York, fifty miles north. We think areas corresponding to the existing British Medical Association areas would be better. At all events, for counties in which there is a scattered county area and a large number of county boroughs. . . The number of divisions being based on the chief centres of population."

The action of the Sussex practitioners is evidently taken in accordance with a resolution of the Federating Council to the effect that it be the policy of the Union to promote the formation of Non-Panel Associations on the basis of Insurance areas. The suggestion made by Dr. Trotter, however, obviously will require careful consideration.

### Manchester Medical Union.

The National Medical Union of Manchester held its annual meeting on December 17 last. The meeting formally agreed to federate under the National Medical Union. The local Association will in future be known as the Manchester Medical Union. The terms of membership exclude the admittance of any but "non-service" medical practitioners. Those who joined the Union previous to November 29, 1913, can retain their membership by payment of the annual subscription (10s. 6d.) and the federation fee (£1 1s.) for 1914. Medical men residing in any portions of the United Kingdom may become members.

The efforts of the Manchester Union will be chiefly directed towards promoting the non-panel cause locally, and supporting the policy of the National Medical Union in every possible way.

It was also resolved "that the Central Council of the National Medical Union shall be asked to specially consider the cases of those members of the medical profession whose partners are upon the panel, but who do not see their partner's patients, and whose deeds of partnership were in existence before the panels were formed."

It was felt that in certain cases such men might be allowed to join the Union. A resolution passed at the Conference of November 29 at present bars them out.

G. A. Wright, Esq., F.R.C.S., was re-elected President of the Manchester Medical Union; William Coates, Esq., C.B., D.L., Chairman; J. S. Prowse, M.B., Hon. Secretary; E. W. Floyd, Esq., M.B., Hon. Treasurer.

The following are members of the Executive Committee: Drs. Hart, Wilson, Lowe, Stenhouse, Trotter, Woodcock, Westmacott, Malin, Pinder, Clarke, Sarjant, McMillan, Bell, Sawyers, Scott, Mackler, and Grange.

It was decided that, "in the opinion of the general meeting of the Manchester Medical Union, the question of policy should be considered at once, and suggestions submitted to the various amalgamated bodies for consideration; and that in this connection the system of administration of medical benefits adopted by the National Deposit Friendly Society and the Scottish Clerks' Association should be gone into very carefully."

---

Dr. Porter writes from Edinburgh:—

One aspect of the Insurance Act—and the most important—namely, the sociological—has received but scanty consideration. At the time of the Act's inception so much prominence was unfortunately given to the financial side that the sociological was to a great extent obscured. I do not believe that the most optimistic panel doctor could truthfully say that he is as free as he was prior to accepting service under the Act. If the German Insurance struggle is a foretaste of what will happen in this country (and there are many reasons to suppose that it will be) the outlook is a gloomy one for the freedom of the panel doctor.

On a large percentage of the insured population the Act will have a baneful influence. Money easily got is easily spent. I have observed already that the thirty shillings maternity fee does not encourage the father to pay his doctor's bill any sooner, but in certain cases it has induced a slackness amounting to forgetfulness which did not previously exist.

